# Bayesian reasoning with ifs and ands and ors

**DOI:** 10.3389/fpsyg.2015.00192

**Published:** 2015-02-25

**Authors:** Nicole Cruz, Jean Baratgin, Mike Oaksford, David E. Over

**Affiliations:** ^1^Department of Psychological Sciences, Birkbeck, University of London, London, UK; ^2^Laboratory CHArt (PARIS), Université Paris 8, Paris, France; ^3^Institut Jean Nicod, Paris, France; ^4^Department of Psychology, Durham University, Durham, UK

**Keywords:** uncertain reasoning, deduction, conditionals, coherence, conjunction fallacy

## Abstract

The Bayesian approach to the psychology of reasoning generalizes binary logic, extending the binary concept of consistency to that of coherence, and allowing the study of deductive reasoning from uncertain premises. Studies in judgment and decision making have found that people’s probability judgments can fail to be coherent. We investigated people’s coherence further for judgments about conjunctions, disjunctions and conditionals, and asked whether their coherence would increase when they were given the explicit task of drawing inferences. Participants gave confidence judgments about a list of separate statements (the statements group) or the statements grouped as explicit inferences (the inferences group). Their responses were generally coherent at above chance levels for all the inferences investigated, regardless of the presence of an explicit inference task. An exception was that they were incoherent in the context known to cause the conjunction fallacy, and remained so even when they were given an explicit inference. The participants were coherent under the assumption that they interpreted the natural language conditional as it is represented in Bayesian accounts of conditional reasoning, but they were incoherent under the assumption that they interpreted the natural language conditional as the material conditional of elementary binary logic. Our results provide further support for the descriptive adequacy of Bayesian reasoning principles in the study of deduction under uncertainty.

## INTRODUCTION

Most everyday and scientific inferences are from uncertain premises, with the aim of forming and revising beliefs and making decisions. For example, some hypotheses about global warming are more highly confirmed than others, but all are uncertain to some degree, and yet there have to be inferences from these hypotheses to further scientific research and practical decision making. Given the ubiquity of reasoning under uncertainty, an important question in the psychology of reasoning is how good people are at it, and what can improve it when it falls short of the appropriate normative theory.

Tversky and Kahneman (1983) pointed out that “…the normative theory of judgment under uncertainty has treated the coherence of belief as the touchstone of human rationality.” *Coherence* is the normative foundation of the Bayesian approach to the study of cognition (Chater and Oaksford, 2008), which is having an immense impact on the psychology of reasoning (Elqayam and Over, 2013). To be coherent is to conform to the axioms of probability theory, which are justified by the Dutch book theorem (de Finetti, 1974).

There are tasks and contexts in which there appears to be a remarkably good correspondence between people’s probability judgments and probability theory (Griffiths and Tenenbaum, 2006; Oaksford and Hahn, 2007; Fiser et al., 2010; Oaksford and Chater, 2013). But there are also contexts in which people are incoherent. Until very recently, there were only limited studies of whether people are coherent in their judgments about the basic logical connectives of conjunction, disjunction, and the conditional. Of course, there have been innumerable papers on the *conjunction fallacy* (Tversky and Kahneman, 1983): judging that the probability of a conjunction, *P(p and q)*, is greater than the probability of one of its conjunctions, *P(p)*. The valid logical inference related to this fallacy is *and-elimination*: inferring *p* from *p and q*. But this is just one out of many logical inferences in which conjunction occurs. There have been relatively few studies of the *disjunction fallacy* (Bar-Hillel and Neter, 1993): judging that *P(p)* is greater than *P(p or q)*. The valid inference for this fallacy is *or-introduction*: inferring *p or q* from *p*. There should be wider studies of probability judgments about conjunctions and disjunctions, especially when these connectives are related to the conditional, *if p then q*, since conditionals are at the heart of so much reasoning in both everyday affairs and science.

The purpose of this paper is to extend the study of whether people’s probability judgments about conjunctions, disjunctions, and conditionals are coherent. Our approach is that of the new paradigm in the psychology of deductive reasoning, which goes beyond the binary distinction between categorical belief in the truth, or falsity, of propositions to the full range of degrees of belief, or subjective probabilities (Evans and Over, 2004, 2013; Oaksford and Chater, 2007, 2012, 2013; Pfeifer and Kleiter, 2010, 2011; Baratgin et al., 2013; Pfeifer, 2013; Over, in press). The probabilistic approach has taken two important steps in the study of deduction: (1) it represents uncertainty in the premises and conclusions of inferences, and (2) it represents the probability of the natural language indicative conditional, *P(if p then q)*, as the conditional probability of *q* given *p*, *P(q|p)*. The relation, *P(if p then q)* = *P(q|p)*, is so fundamental for a Bayesian account of conditional reasoning that it has simply been called *the Equation* (Edgington, 1995; Oaksford and Chater, 2007). A conditional that satisfies the Equation has been called the *probability conditional* (Adams, 1998; Oaksford and Chater, 2007), but we call it here the *conditional event* (following de Finetti, 1995). The conditional probability in the Equation, *P(q|p)*, is not defined by the ratio *P(p and q)*/*P(p)* in our approach (see also Pfeifer, 2014). One can easily think of cases in which people have a clear degree of belief about *P(q|p)* even though they judge that *P(p)* = 0, or they cannot make a judgment at all about *P(p)* (Adams, 1998). We rather argue that people infer the conditional probability in a *Ramsey test*, that is, a mental simulation in which they hypothetically suppose *p* to be the case, make whatever changes to their beliefs are necessary to preserve consistency, and assess the probability of *q* on this basis (Stalnaker, 1968; Ramsey, 1994; Evans and Over, 2004). Both (1) and (2) have received strong and converging empirical support (Oaksford et al., 2000; Evans et al., 2003; Oberauer and Wilhelm, 2003; Oberauer et al., 2007; Over et al., 2007; Douven and Verbrugge, 2010; Politzer et al., 2010; Fugard et al., 2011b; Baratgin et al., 2014; Cruz and Oberauer, 2014; Singmann et al., 2014).

In an influential alternative approach, mental model theory, the natural language indicative conditional is taken to have the same full models as the *material conditional* of elementary extensional logic, which is logically equivalent to *not-p or q*. The material conditional is truth functional, that is, its truth or falsity is a function of the truth or falsity of its elementary components, the propositions *p* and *q*. It is false when its antecedent *p* is true and the consequent *q* is false, and it is true in the other three possible cases (that is, the cases *p and q*, *not-p and q*, and *not-p and not-q*). In mental model theory, *P(if p then q)* = *P(not-p or q)* is supposedly the correct normative probability judgment to make (Johnson-Laird and Byrne, 1991, 2002; Byrne and Johnson-Laird, 2009, 2010). Whether *P(if p then q)* equals *P(q|p)* or *P(not-p or q)* very much affects which judgments are coherent in conditional reasoning, as we will see below.

Consider the uncertain premises and possible uncertain conclusions that form the basis of most of our ordinary and scientific reasoning. The axioms of probability theory can be used to determine whether combinations of these premises and conclusions are, or are not, coherent (for recent examples see Pfeifer and Kleiter, 2005, 2009; Gilio and Over, 2012). For instance, there are the valid inferences of *and-elimination*, referred to above, and also *and-introduction*: inferring *p and q* from the separate premises *p* and *q*. For probability judgments about *p*, *q*, and *p and q* to be coherent, *P(p and q)* must lie in the interval between P(p) + P(q) – 1 (or 0 if this sum is negative) at the lower end, and the minimum of *P(p)* and *P(q)* at the upper end (Pfeifer and Kleiter, 2005). For example, *P(p)* = *P(q)* = 0.6 and *P(p and q)* = 0.1 is incoherent because *P(p and q)* is too low, and *P(p)* = *P(q)* = 0.6 and *P(p and q)* = 0.7 is incoherent, and the conjunction fallacy is committed, because now *P(p and q)* is too high. Our question in this paper is whether people are generally coherent in their conjunctive, disjunctive, and conditional premises and conclusions, and whether their coherence is improved when they are given explicit inferences. Tversky and Kahneman (1983) did not ask their participants to infer degrees of confidence in the conclusion *p* from an uncertain *p and q* premise in an explicit inference, but we did ask participants in our experiments.

Studies of the coherence between premises and conclusions of people’s reasoning has only just begun (Pfeifer and Kleiter, 2005, 2010, 2011; Pfeifer, 2013; Politzer and Baratgin, under review; Singmann et al., 2014; Evans and Over, under review). There is evidence, for example, that people are coherent in explicit *and-introduction* inferences (Pfeifer and Kleiter, 2005; Politzer and Baratgin, under review). There are also some studies of the classical conditional inferences of modus ponens (MP), modus tollens (MT), affirmation of the consequent (AC), and denial of the antecedent (DA), and it has been found that the degree to which people are coherent can increase when they are given some of these conditional inferences as explicit tasks to perform (Evans and Over, under review; see also Pfeifer and Kleiter, 2010; Singmann et al., 2014).

We conducted two experiments focusing on conjunctions, disjunctions, and their relationships with conditionals, and comparing probability judgments about the premises and conclusions when these were given as separate statements and when they were arranged as explicit inferences. Experiment 1 looked at inferences between disjunctions and conditionals, and Experiment 2 at inferences between conjunctions and conditionals. The inferences are summarized in Table 1.

**TABLE 1:** **The inferences used in Experiments 1 and 2**.

	**Experiment 1**		**Experiment 2**
1.1	p, therefore p or q	2.1	p & q, therefore if p then q
1.2	not-p, therefore not-p or q	2.2	p, q, therefore if p then q
1.3	If p then q, therefore not-p or q	2.3	p & q, therefore p
1.4	if not-p then q, therefore p or q	2.4	p & q, therefore q
1.5	p or q, therefore if not-p then q		
1.6	not-p or q, therefore if p then q		

Inferences 1.1 and 1.2 are logically equivalent, as are inferences 1.3 and 1.4, as well as inferences 1.5 and 1.6. They differ only in the position of the negation they contain. The two positions of the negation instantiated in the inferences are those for which the largest negation effects have been reported in the literature (Oberauer et al., 2011; Espino and Byrne, 2013). We introduced this variation in order to control for negation effects. Experiment 1 assessed two further inferences, and Experiment 2 six further inferences, which are not listed in Table 1. These additional inferences were used to investigate other questions, and are not discussed here further.

Inferences 1.1 and 1.2 are logically equivalent forms of *or-introduction*, and here it is clearly incoherent to judge that the probability of the conclusion is lower than that of the premise. It is a consequence of the axioms of probability theory that *P(p)* ≤ *P(p or q)*. In the binary approach, it is inconsistent to assume the truth of the premise of *or-introduction*, *p*, but not to accept that *p or q* follows. Binary studies found that people did endorse this inference at just above chance levels, but also that there was significant resistance to it (Rips, 1983, 1994; Braine et al., 1984). This finding has generally been explained as a pragmatic effect: people are unwilling to draw the inference because it would be misleading in a conversation with another person to endorse *p or q* when one can make the more informative statement *p* (Grice, 1989; see also Bar-Hillel and Neter, 1993; Tversky and Koehler, 1994; Fugard et al., 2011a). The much wider Bayesian approach can cover the special case of binary inconsistency by letting a probability of 1 represent “true” and a probability 0 represent “false.” The binary findings could be said to reveal implicit incoherent reasoning because people are, in effect, making *P(p)* = 1, *p* is “true,” and *P(p or q)* = 0, *p or q* is “false.” However, we predicted greater coherence when people are explicitly asked for their degrees of belief about *p* and *p or q*. People can then state their degrees of belief directly, without needing to consider additional pragmatic factors that arise when communicating with another speaker. This should lead to the prevention, or at least to a strong reduction, of pragmatic effects.

Inferences 1.3 and 1.4 are logically equivalent *if-to-or* inferences and go from a conditional to a disjunction. Supposing *if p then q* is equivalent to the material conditional, *P(if p then q)* = *P(not-p or q)*, any other judgment is incoherent. Supposing *if p then q* is the conditional event, *P(if p then q)* = *P(q|p)*. It follows from the axioms of probability theory that *P(q|p)* ≤ *P(not-p or q)*, and probability judgments must conform to this relation to be coherent.

Inferences 1.5 and 1.6 are logically equivalent *or-to-if* inferences and go from a disjunction to a conditional. If the conditional in these inferences is interpreted as the material conditional, then the same equivalence holds as for 1.3 and 1.4, and judgments are only coherent when the premise and conclusion are assigned the same probability. If the conditional is interpreted as the conditional event, then judgments are coherent when they conform to the relation *P(q|p)* ≤ *P(not-p or q)*. Thus the relation that must hold for the inferences to be coherent is the same for 1.3–1.4 and for 1.5–1.6. The difference is that in the first two the conditional is the premise, and in the second two the conditional is the conclusion. This implies that, if one interprets the conditional as the conditional event, the *if-to-or* inference is coherent when the probability of the conclusion is equal or higher than that of the premise, whereas the *or-to-if* inference is coherent when the probability of the conclusion is equal or lower than that of the premise. This difference in the conditions for coherence of the two inferences is reflected in the fact that under a conditional event interpretation, the *if-to-or* inference is valid, whereas the *or-to-if* inference is invalid and can even be a quite a weak inference.

When we speak of validity in this context of uncertain inference, we mean probabilistic validity, or *p-validity*. P-validity is a generalization of binary validity to reasoning under uncertainty. Just as an inference is binary valid when there are no cases in which the conclusion is false and the premise is true, a single premise inference is p-valid when there are no coherent cases in which the probability of the conclusion is lower than the probability of the premise (see Adams, 1998, on p-validity for inferences with more than one premise; Singmann et al., 2014; Evans and Over, under review, for applications in the psychology of reasoning). For the *or-to-if* inference, such cases are possible. Consider an instance of 1.5. We might have a high degree of confidence that our bicycle is outside our apartment in Paris where we left it. That should, if we are coherent in the *or-introduction* inference, give us a high degree of confidence that our bicycle is outside our apartment in Paris or in Timbuktu. But we do not have any confidence that, if our bicycle is not outside our apartment in Paris, then it is in Timbuktu. It is much more reasonable to infer that, if our bike is not there, it is somewhere else in Paris after being stolen. Johnson-Laird and Byrne (2002, p. 650) claimed that people always endorse 1.5, but Gilio and Over (2012) have an analysis of when 1.5 and 1.6 are, and are not, reasonable inferences to make, and Over et al. (2010) have supporting results. Because the question of whether people’s responses to the *or-to-if* inferences are coherent depends on how the conditional is interpreted, our investigation of these inferences does more than reveal their coherence in general. It also tells us about the modal interpretation of the conditional. If people’s judgments are highly incoherent for one interpretation, and yet highly coherent for another, there is an argument in favor of the interpretation that renders their judgments coherent.

Inferences 2.1 and 2.2 are from a conjunction to the conditional. The first has the conjunction as a single premise, whereas the second has the two conjuncts as separate premises. It is easiest to state what is coherent for the single premise inference to the conditional event. By probability theory, *P(p and q)* = *P(p)P(q|p)* ≤ *P(q|p)*. The formula for the coherence of judgments about the premises and conclusion of the 2.2 inference is more complex because it requires taking into account that the premises can covary to different degrees (Kleiter, 2014). The formula for it is reported in Experiment 2 below. Because of this additional complexity in processing coherence for inference 2.2, we wanted to assess whether people’s responses complied with coherence more often for 2.1 than for 2.2.

Inferences 2.3 and 2.4 are forms of *and-elimination*, and we have already stated above how coherence is determined for them. Not conforming to coherence for this inference is to commit the conjunction fallacy. We therefore wanted to test for this case whether our general prediction holds: that people’s probability judgments more often conform to coherence when they are given the explicit task of drawing inferences.

## EXPERIMENT 1

### METHODS

#### Participants

A total of 1140 participants from English speaking countries completed the online experiment, in exchange for €0.1. From this initial sample we excluded cases that had the same IP address as a previously recorded participant, cases that provided the same response on all trials, and cases that had a reported age below 12 or above 100^1^. The final sample consisted of 871 participants. Their mean age was 35 years (range 12–78). They reported different levels of formal educational training, and 87% reported having “good” or “very good” English language skills.

#### Material and design

Participants were shown a short scenario describing a person, and then presented with a series of statements about the person. The statements either appeared one at a time on the screen, in random order for each participant in the *statements group*, or the statements were presented in pairs as the premises and conclusions of explicit inferences in the *inferences group*. Participants in the statements group were asked to judge how confident they were in each statement, by typing in a percentage between 0% (“no confidence at all”) and 100% (“complete confidence”). Participants in the inferences group were asked to judge how confident they were in the premise of the argument, and then how confident they were in the conclusion, given the premise. Participants in the inferences group used the same percentage scale as those in the statements group to provide their answers.

Two scenarios were varied between participants: The Linda scenario (Tversky and Kahneman, 1983), with the standard description of Linda, and a scenario describing a person conforming to a stereotype quite unlike that of Linda. Below is a sample trial in the statements group and in the inferences group, using the Linda scenario:

Statements group:

Now consider the following statement about Linda:

Please indicate how much confidence you would have in this statement. Please give a percentage rating from 0% (no confidence at all) to 100% (complete confidence).

“Linda votes for the Labour Party or the Green Party”

Inferences group:

Now consider the following argument about Linda:

Next to A please indicate how much confidence you would have in the premise of the argument. Next to B please indicate how much confidence you would have in the conclusion, given the premise. Please give a percentage rating from 0% (no confidence at all) to 100% (complete confidence).

A. “Linda votes for the Labour Party or the Green Party”

B. “Therefore, if Linda does not vote for the Labour Party, then she votes for the Green Party”

In the inferences group, participants judged each inference twice with different contents. The allocation of scenario contents to inferences was counterbalanced across participants, leading to eight different booklets, four for each scenario. In the statements group, each participant rated the entire set of contents created for the relevant scenario, leading to two booklets, one for each scenario. In order to compensate for the difference in sample size between groups resulting from the different number of booklets in each group, we placed a weight on the otherwise random procedure for assigning participants to booklets, such that participants were twice as likely to receive any one of the booklets of the statements group than any one of the booklets of the inferences group. This resulted in sample sizes of *n* = 305 and *n* = 566 for the statements and inferences group, respectively.

#### Procedure

The experiment took place online using the platform Crowd-Flower. On the first screen participants viewed the instructions and a sample trial. The next screen showed the scenario within which the statements, or respectively the inferences, were to be assessed. These then followed, presented one at a time on the screen. A further screen asked for demographical information, and a final screen provided debriefing information. The whole procedure took on average 4.24 min for the statements group and 5.23 min for the inferences group.

### RESULTS

We measured above chance compliance with coherence using a method introduced by Evans et al. (under review; see also Pfeifer and Kleiter, 2009). First, we computed the difference between the probability assigned to the conclusion and the probability assigned to the premise. We then computed a binary variable to encode whether this difference indicated that the response was coherent or not. Thus, for or-introduction, 1.1–1.2, and the *if-to-or* inferences, 1.3–1.4, this variable took the value 1 when the difference was positive or 0, and took the value 0 otherwise. For the *or-to-if* inferences, 1.5–1.6, the variable took the value 1 when the difference was 0 or negative, and took the value 0 otherwise. This computation was performed separately for each participant and inference. We call this variable *observed coherence*. Next, we computed the probability of a response being coherent by chance, *chance coherence*. On the assumption that a random response can fall equally likely on any point of the probability scale, the probability of complying to coherence by chance corresponds to the width of the coherence interval. This is a simplifying assumption because there is evidence that people’s probability estimates might be biased at the boundaries of the interval, in a way that could lead to higher chance rates for extreme cases (c.f. Stewart et al., 2006). However, we considered a uniform distribution of chance rates a sufficiently accurate approximation to allow an assessment of the hypotheses at hand. On this assumption, if a person assigns for instance a probability of 0.6 to the premise of an *or-introduction* inference, then the probability she assigns to the conclusion is coherent if it falls within the interval between 0.6 and 1. Because the width of this interval is 0.4, the chance rate of conforming to coherence is in this case also 0.4. Finally, we subtracted chance coherence from observed coherence, to obtain a measure of the extent to which responses were coherent at levels above those expected by chance, *above chance coherence*.

**FIGURE 1:**
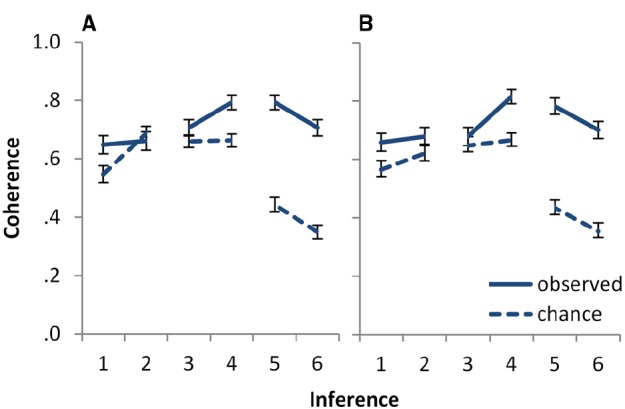
**Observed versus chance coherence for the six inferences of Experiment 1, (A) for the statements and (B) for the inferences task.** Inferences 1 and 2 are logically equivalent *or-introduction* inferences, with a negation absent in 1 and present in the premise of 2. Inferences 3 and 4 are logically equivalent *if-to-or* inferences. Inference 3 has a negation in the conclusion and inference 4 in the premise. Inferences 5 and 6 are logically equivalent or-to-if inferences. Inference 5 has a negation in the conclusion and inference 6 in the premise. See Table 1 for the precise logical form of the inferences. Error bars show 95% CI.

The ratings of above chance coherence were submitted to a mixed ANOVA with the between subjects factor of *task* (statements, inferences) and the within subjects factor of *inference* (*or-introduction*, *if-to-or*, and *or-to-if*). Throughout the paper, the Greenhouse–Geisser correction of degrees of freedom for lack of sphericity was used when appropriate, and the Bonferroni–Holm correction of *p*-values for multiple comparisons was used to define the limit of a significant effect, while reporting the original *p*-values. The results are depicted in Figure 1. The overall intercept was significant, *F*(1,869) = 885.29, *p* < 0.001, ${\text{η}}_{\text{p}}^{\text{2}}$ = 0.505, indicating that overall probability judgments were coherent at above chance level. There was also a main effect of inference, *F*(1.382,1201.390) = 266.28, *p* < 0.001, ${\text{η}}_{\text{p}}^{\text{2}}$ = 0.235: above chance coherence differed for the three inferences. No other effects were significant (highest *F* = 1.07, lowest *p* = 0.30). In particular, there was no significant effect of task.

Follow-up analyses of the effect of inference showed that although responses were coherent at above chance level for all three inferences, the degree of above chance coherence was higher for *or-to-if* than for *if-to-or*, *F*(1,869) = 270.99, *p* < 0.001, ${\text{η}}_{\text{p}}^{\text{2}}$ = 0.238; and higher for *if-to-or* than for *or-introduction*, *F*(1,869) = 16.50, *p* < 0.001, ${\text{η}}_{\text{p}}^{\text{2}}$ = 0.019. Thus, responses to both *or-to-if* and *if-to-or* were consistently above chance. Responses to *or-introduction* were also coherent more often than expected by chance, although somewhat less often than responses to the other two inferences. An inspection of Figure 1 suggests that the difference between the *if-to-or* and the *or-introduction* inferences was due mainly to the lower coherence for *or-to-if* for inference 1.2 in the statements task. In line with this, a comparison between the two inferences restricted to the inference task showed no difference in above chance coherence between the two, *F*(1,565) = 1.85, *p* = 0.17, ${\text{η}}_{\text{p}}^{\text{2}}$ = 0.003.

We conducted a further analysis of the *or-to-if* inference in which we excluded responses that are coherent for both the conditional event and the material conditional interpretation of the conditional: responses that assigned the same probability to the premise and conclusion. We treated as coherent only those responses that are coherent for a conditional event interpretation: responses that assigned a lower probability to the conclusion than to the premise. On a material conditional interpretation, the only coherent response to this inference is to assign the same probability to both the premise and conclusion, and assuming that people interpret the conditional as the material conditional, the mean difference between premise and conclusion probability would be expected to be 0. There might be some scattering of probabilities above and below 0, but no systematic drift in any direction. We would expect there to be no effect of coherence for this analysis. On a conditional event interpretation, responses are coherent when the probability of the conclusion of the *or-to-if* inference is equal to or lower than that of the premise. On this interpretation, we would expect coherence to be lower for this analysis than for the analysis using all the data, because a subset of coherent responses would not be considered. The absence of an effect of coherence would also be compatible with this interpretation, and would then render the analysis uninformative to the question at hand. However, a remaining effect of coherence in the expected direction would constitute specific evidence for a conditional event interpretation and against a material conditional interpretation of the conditional.

An univariate ANOVA on above chance coherence for the *or-to-if* inference, using only the data for which probability judgments differed for premise and conclusion in each individual case, yielded a significant intercept, *F*(1,362) = 100.27, *p* < 0.001, ${\text{η}}_{\text{p}}^{\text{2}}$ = 0.217: responses to *or-to-if* were coherent at levels above chance when only considering as coherent those responses that are coherent for the conditional event and incoherent for the material conditional interpretation of the conditional.

Although Figure 1 shows the results separately for each position of the negation, we did not find any consistent effects regarding this variable. We also did not have any hypotheses about it, but introduced it only as a control variable, to be able to obtain a pattern of results that could be generalized across positions of the negation.

### DISCUSSION

We investigated the extent to which people’s probability judgments were coherent for the premises and conclusions of inferences 1.1 to 1.6, when these were separate statements, in the statements group, and when they were formed into explicit inferences, in the inference group. We found people’s responses to be coherent at levels above chance for the three inferences forms investigated, 1.1–1.2, 1.3–1.4, and 1.5–16, in both the statements group and the inferences group. There was therefore clear evidence that people’s probability judgments conform to Bayesian principles, and at the same time there was no evidence that this conformity was improved further in the context of explicit inference.

Responses for the *or-introduction* inferences, 1.1–1.2, were found to be coherent at levels above chance, and to a degree similar to that for the *if-to-or* inferences 1.3–1.4., implying that participants endorse this inference when they are asked for their degrees of belief and not whether, as in a binary experiment, the conclusion necessarily follows given the premise. This finding is in accordance with our prediction that pragmatic factors have less effect on this inference when people are asked for their degrees of belief. Also supporting this conclusion, (Politzer and Baratgin, under review) found, using an ordinal response format for degrees of belief, that responses for *or-introduction* were coherent to a level comparable to five other valid inferences. But they also found coherence rates for the inference to be lower when the premise was certain than when it was uncertain. The limiting case of certainty, which is in effect the only one studied in a binary approach, may give a misleading picture of how far people conform to Bayesian standards, and this hypothesis will have to be investigated further. One option would be through a comparison of responses with binary and with continuous response format. Although a mapping of the two response scales is not straightforward, larger differences between them would still be informative (see Markovits and Handley, 2005; Singmann and Klauer, 2011, for two ways of carrying out such a comparison).

The analysis of responses to the *or-to-if* inferences, 1.5–1.6, showed that participants’ responses would fail to be coherent at levels above chance under a material conditional interpretation of the conditional, *P(if p then q)* = *P(not-p or q)*, whereas they would be coherent at levels above chance if the natural language conditional is interpreted as the conditional event, *P(if p then q) = P(q|p)*. There does not appear to be any reason why people would be so highly incoherent for these inferences if they had a material conditional understanding of the natural language conditional. But if they have a conditional event understanding, our finding is to be expected, and it provides new support for the conditional event interpretation of the conditional. People’s conditional reasoning can be much “improved” from a Bayesian point of view, if their understanding of the conditional is, to begin with, correctly identified by the psychology of reasoning.

Responses to the *if-to-or* inferences were likewise reliably coherent above chance levels, showing that participants respected the difference in the coherence conditions between these and the *or-to-if* inferences.

Experiment 1 investigated inferences between conditionals and disjunctions. Our second experiment addresses inferences between conditionals and conjunctions, and includes the content that is famous for causing the incoherence of the conjunction fallacy.

## EXPERIMENT 2

### METHOD

#### Participants

Forty-eight students from the University of Orsay, France, took part in the experiment on a voluntary basis. Their mean age was 20 years (range 18–24). They had different majors, although the majority studied biology or medicine. All participants were French native speakers.

#### Material and design

The material and design were very similar to those of Experiment 1. However, only the Linda scenario was used, and because the original inferences contained no negations, no negation effects were assessed. Inferences 2.1 and 2.2, *and-to-if* forms, used contents prototypical for the scenario, in order to obtain higher probability estimates for the premises and thus lower probabilities of conforming to coherence just by chance. Inferences 2.3 and 2.4, *and-elimination* inferences, varied the prototypicality of the content for the scenario in the same way as in Tversky and Kahneman’s (1983) original work on the conjunction fallacy. To take an example from the explicit inferences group, participants read the standard description of Linda and were then asked to state what confidence they had in “Linda is banker” as a conclusion explicitly inferred from “Linda is a feminist and a banker” as a premise.

Participants were divided into two groups of equal size. The booklets for the statements group contained a continuous list of statements. In the booklets for the inferences group, each inference appeared on a separate page. Four booklets were constructed for each group, which differed only in the order in which the items were presented.

#### Procedure

Participants were tested in the university library in small groups of up to four participants. They worked at their own pace, and took 10 to 15 min to complete.

### RESULTS

Responses to inference 2.1 are incoherent when the probability assigned to the conclusion is lower than that of the premise. For inference 2.2, the computation of coherence is more complicated because it takes into account the minimum and maximum overlap between the two premises. The lower and upper coherence bounds for this inference, when the entailed conditional is interpreted as the conditional event, are as follows:

$$P\left(\mathrm{conclusion}\right)=P\left(q|p\right)\in \left[\max\left\{0,\frac{p+q-1}{p}\right\},\min\left\{\frac{q}{p},1\right\}\right]$$

We only computed coherence for the conditional event interpretation of the conditional. However, the coherence bounds for this interpretation are stronger than those for the material conditional. Therefore, any response that is coherent for the above interpretation is also coherent for the material conditional interpretation. See Politzer (2014) for a proposal of how to obtain the intervals of coherence for a wide range of inferences in an intuitive way using a water tank analogy.

**FIGURE 2:**
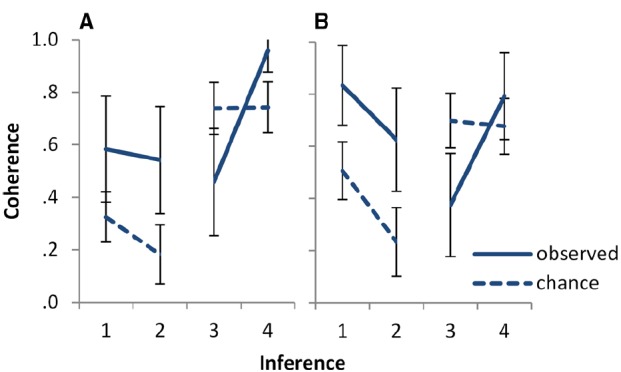
**Observed versus chance coherence for the four inferences of Experiment 2, (A) for the statements and (B) for the inferences task.** Inferences 1 and 2 are *and-to-if* inferences. The first has the conjunction *p and q* as single premise, the second has *p* and *q* as two separate premises. Inferences 3 and 4 are *and-elimination* inferences. The first has prototypical, and the second counter-prototypical content for the scenario. See Table 1 for the precise logical form of the inferences. Error bars show 95% CI.

The results are illustrated in Figure 2. To assess whether the additional complexity of processing coherence for 2.2 as compared to 2.1 leads to higher levels of above chance coherence for 2.1, we conducted a mixed ANOVA on above chance coherence with the between subjects factor of task (statements, inferences) and the within subjects factor of inference (2.1, 2.2). The intercept was significant, *F*(1,46) = 36.83, *p* < 0.001, ${\text{η}}_{\text{p}}^{\text{2}}$ = 0.445, indicating that participants’ responses were coherent to a degree above that expected by chance. No other effects were significant (largest *F* = 1.03, lowest *p* = 0.32). In particular, there was no significant effect of task or of inference.

To assess whether the conjunction fallacy is reduced in the context of an inference task, a mixed ANOVA on above chance coherence for inferences 2.3 and 2.4 was conducted, with task as a between subjects factor and inference as a within subjects factor. There was a main effect of inference, *F*(1,46) = 33.31, *p* < 0.001, ${\text{η}}_{\text{p}}^{\text{2}}$ = 0.420: The two inferences differed in above chance coherence. No other effects were significant (largest *F* = 2.12, smallest *p* = 0.15). In particular, the intercept was not significant, indicating that overall the coherence of participants’ judgments for these inferences did not differ from chance; and there was no effect of task. In line with the pattern in Figure 2, follow up analyses indicated that coherence was above chance for 2.3, which used prototypical content, *F*(1,46) = 10.23, *p* = 0.002, ${\text{η}}_{\text{p}}^{\text{2}}$ = 0.182; and coherence was below chance for 2.4, which used counter-prototypical content, *F*(1,46) = 18.37, *p* < 0.001, ${\text{η}}_{\text{p}}^{\text{2}}$ = 0.285.

### DISCUSSION

The finding of above chance coherence for the *and-to-if* inferences 2.1 and 2.2 extends the evidence of Experiment 1 to inferences relating conjunctions and conditionals. The absence of an effect of inference implies that at least for the materials used, the additional requirement in 2.2 of integrating two premise probabilities did not reduce the coherence of people’s responses. The absence of an effect of task suggests that above chance coherence for this inference was also not affected by the presence of an explicit inference task, similar to the findings from Experiment 1. A further investigation of the extent to which the requirement of integrating premise probabilities affects people’s reasoning performance could vary the degree of overlap between premises, as well as the assessment of additional indicators of task difficulty, such as response times.

People’s responses to the *and-elimination* inferences 2.3 and 2.4 were coherent at levels above chance, when the materials did not have the content that caused the conjunction fallacy in Tversky and Kahneman (1983). This result is in line with other findings in the probabilistic approach using different methodologies (Pfeifer and Kleiter, 2005; Politzer and Baratgin, under review). However, when the material did have the content known to cause the fallacy, participants were incoherent, just as Tversky and Kahneman would predict for our statements group. Tversky and Kahneman did not predict whether the fallacy would be found when *p* (or *q*) was explicitly inferred from *p and q* as a premise. Stating a degree of confidence in the conclusion of such an explicit inference could arguably qualify as what they called a “transparent” problem, to which people should give a coherent answer. Nevertheless, the participants in our inference group were also incoherent by committing the conjunction fallacy, which at least reinforces Tversky and Kahneman’s view of it as a deep fallacy that is hard to overcome.

## GENERAL DISCUSSION

With the advent of the Bayesian approach in the psychology of reasoning, it has become possible to investigate people’s deductive reasoning from uncertain premises, and to assess the extent to which it is coherent. We investigated this topic in two experiments using inferences between conjunctions, disjunctions, and conditionals. We also looked at whether an explicit inference task increases people’s coherence, and examined a number of more specific hypotheses for the individual inferences. People’s probability judgments were coherent at levels above chance for almost all the inference forms investigated. The one exception was when the materials for the *and-elimination* inference were of the content known to cause the conjunction fallacy. The participants, who read the standard description of Linda, were incoherent in their judgments about “Linda is a feminist and a banker” and “Linda is a banker,” even when they inferred the later statement from the former in an explicit inference.

People were generally coherent, complying with the axioms of probability theory, not only in the explicit inference task, but to an equal extent when the task was to evaluate the single statements that formed the inferences one at a time in random order. This absence of an effect of task was not expected. On the one hand, it does provide some support for the descriptive adequacy of the principle of coherence, because it increases the generality of its scope. It stands in accordance with findings on good conformity to Bayesian principles in domains outside of reasoning, where tasks are carried out in a more implicit way, like perception and language comprehension (Fiser et al., 2010; Hsu et al., 2011). And it suggests that addressing the question of what improves Bayesian reasoning should not make us lose sight of the many contexts in which conformity to Bayesian principles is already quite good.

On the other hand, it remains a plausible hypothesis that explicit inference can be an effective use of cognitive resources to improve coherence, to the benefit of reasoning and decision making. The inference forms we considered here, for conjunctions, disjunctions, and their relations to conditionals, may generally be too simple for an effect to be found. Evans et al. (under review) did find that an explicit inference task could increase coherence in a study of MP, MT, AC, and DA. One possibility is that these two-premise conditional inferences require a more complex integration of premise probabilities, and people could be helped to achieve this in explicit inference tasks.

Another possibility is that it was generally easier in the experiment of Evans and et al. (under review) to detect an increase in above chance coherence because the mean probability estimates given to the premises in their experiment were generally higher than in our experiments. Generally, the higher the probability of the premises, the lower the chance rate of coherence and thus the easier it becomes to detect above chance coherence when it is there. This relation holds for MP, MT, AC, and DA, and all the inferences investigated here except for the *or-to-if* inferences, 1.5–1.6, in Experiment 1. For 1.5–1.6, the opposite relation holds: the chance rate of coherence becomes lower, and the probability of detecting above chance coherence larger, the lower the probability assigned to the premise. Because the mean probability ratings for the premises of the inferences in Experiment 1 was relatively low, chance rate coherence was lower for 1.5 and 1.6 than for the other two inferences, 1.1–1.2 and 1.3–1.4. This explains the higher ratings of above chance coherence for 1.5–1.6 compared to those for *if-to-or* inferences, 1.3–1.4, in spite of comparable rates of observed coherence for both inferences. This also explains why the effect of above chance coherence was relatively small for 1.1–1.2 and 1.3–1.4 in spite of their sizeable rates of observed coherence.

Overall, the dependence of chance rate coherence on the probabilities assigned to the premises is relevant for the interpretation of the presence or absence of incremental effects of coherence, predicted in this case by the presence of an inference task. But it is also relevant to the interpretation of above chance coherence taken by itself, as well as for the interpretation of differences in above chance coherence between inferences. Future experiments on these questions could therefore aim at more adequate control of the premise probabilities, either by letting them be provided by the experimenter, or by conducting a larger pre-test of materials and selecting those with similar probabilities.

As noted above, the high coherence rates described for 1.5–1.6 *or-to-if* inferences, displayed in Figure 1, depend on the conditional being interpreted as the conditional event. If the natural language conditional corresponded to the material conditional, with *P(if p then q)* = *P(not-p or q)* as implied by mental model theory (Byrne and Johnson-Laird, 2009), then the responses to 1.5–1.6 would be incoherent at levels above chance. These results provide strong evidence for the conditional event interpretation of the conditional, and highlight the importance of taking into account people’s semantic interpretation of the premises and conclusions for assessing how far they conform to Bayesian principles.

The results from Experiment 2 on the *and-elimination* inferences 2.3 and 2.4 demonstrate that above chance coherence for these forms depends on there being no conflict between the probability of a statement and its contextual prototypicality. It is remarkable how slight variations in these factors can lead to incoherent judgments that resist even explicit inferences. It is a challenge to all accounts of the conjunction fallacy to explain why it persists through apparently “transparent” *and-elimination* inferences. The very reliability of this finding highlights the relevance of investigating further what is driving the conjunction fallacy (see Jarvstad and Hahn, 2011; Oaksford, 2013; Pothos and Busemeyer, 2013; Tentori et al., 2013, for a recent discussion). However, it is more remarkable still that when such conflicts are not present, people give generally coherent probability judgments even in the absence of explicit inference tasks, at least for conjunctions, disjunctions, and their relations to conditionals. This provides further evidence of the descriptive adequacy of Bayesian reasoning principles.

### Conflict of Interest Statement

The authors declare that the research was conducted in the absence of any commercial or financial relationships that could be construed as a potential conflict of interest.

## Footnotes

1 A reanalysis of the data excluding the 20 participants with a reported age between 12 and 17 led to the same pattern of significant and non-significant results.
