# Corrigendum: Bayesian reasoning with ifs and ands and ors

**DOI:** 10.3389/fpsyg.2015.00718

**Published:** 2015-05-27

**Authors:** Nicole Cruz, Jean Baratgin, Mike Oaksford, David E. Over

**Affiliations:** ^1^Department of Psychological Sciences, Birkbeck, University of LondonLondon, UK; ^2^Laboratory CHArt (PARIS), Université Paris 8Paris, France; ^3^Institut Jean NicodParis, France; ^4^Department of Psychology, Durham UniversityDurham, UK

**Keywords:** uncertain reasoning, deduction, conditionals, coherence, conjunction fallacy

In the article “Bayesian reasoning with ifs and ands and ors,” by Nicole Cruz, Jean Baratgin, Mike Oaksford, and David E. Over (*Frontiers in Psychology*, 2015, Vol. 6, Art. 192), on page 6, Figure 2, the x-axis in both panels would have to read “1, 2, 4, 3” in order to correctly represent the figure. Rearranging the figure to retain an x-axis labeling in ascending order, the corrected Figure 2 is displayed as follows.

**Figure 2:**
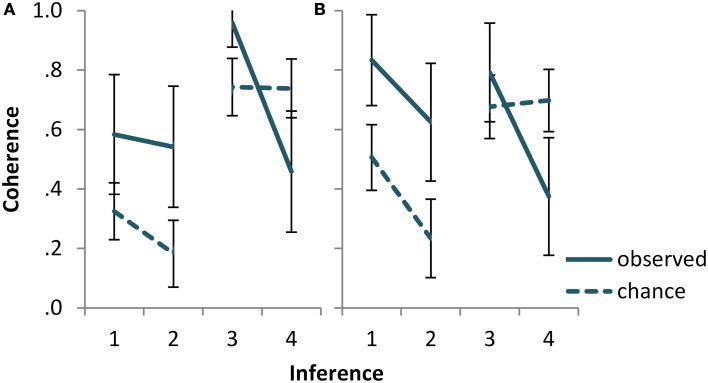
**Observed vs. chance coherence for the four inferences of Experiment2, (A) for the statements and (B) for the inferences task**. Inferences 1 and 2 are *and-to-if* inferences. The first has the conjunction *p and q* as single premise, the second has *p* and *q* as two separate premises. Inferences 3 and 4 are *and-elimination* inferences. The first has prototypical, and the second counter-prototypical content for the scenario. See Table 1 for the precise logical form of the inferences. Error bars show 95% CI.

## Conflict of interest statement

The authors declare that the research was conducted in the absence of any commercial or financial relationships that could be construed as a potential conflict of interest.

